# Expression and characterization of monofunctional alcohol dehydrogenase enzymes in *Clostridium thermocellum*

**DOI:** 10.1016/j.mec.2024.e00243

**Published:** 2024-06-20

**Authors:** Daniela Prates Chiarelli, Bishal Dev Sharma, Shuen Hon, Luana Walravens Bergamo, Lee R. Lynd, Daniel G. Olson

**Affiliations:** a Centro de Biologia Molecular e Engenharia Genética (CBMEG), Universidade Estadual de Campinas (UNICAMP), Campinas, SP, Brazil; b Programa de Pós-Graduação Em Genética e Biologia Molecular, Instituto de Biologia (IB), Universidade Estadual de Campinas (UNICAMP), Campinas, SP, Brazil; c Thayer School of Engineering, Dartmouth College, Hanover, NH, USA; d Center for Bioenergy Innovation, Oak Ridge National Laboratory, Oak Ridge, TN, USA

**Keywords:** *Clostridium thermocellum*, Alcohol dehydrogenase, Heterologous expression, Ethanol

## Abstract

*Clostridium thermocellum* is a thermophilic anaerobic bacterium that could be used for cellulosic biofuel production due to its strong native ability to consume cellulose, however its ethanol production ability needs to be improved to enable commercial application. In our previous strain engineering work, we observed a spontaneous mutation in the native *adhE* gene that reduced ethanol production. Here we attempted to complement this mutation by heterologous expression of 18 different alcohol dehydrogenase (*adh)* genes. We were able to express all of them successfully in *C. thermocellum*. Surprisingly, however, none of them increased ethanol production, and several actually *decreased* it. Our findings contribute to understanding the correlation between *C. thermocellum* ethanol production and Adh enzyme cofactor preferences. The identification of a set of *adh* genes that can be successfully expressed in this organism provides a foundation for future investigations into how the properties of Adh enzymes affect ethanol production.

## Highlights

• 18 monofunctional ADH enzymes were expressed in *C. thermocellum.*
• Enzyme expression was an attempt to complement an *adhE* mutation.
• High levels of NADH-linked ADH activity decreased ethanol production.

## 1 Introduction

*Clostridium thermocellum* is a promising candidate for the production of biofuels from cellulose due to its native ability to consume cellulose (Lynd et al., 2022). This ability is the result of a highly specialized multi-enzyme complex known as a cellulosome (Bayer et al., 2004; Lamed et al., 1983), and this allows for cellulose consumption at rates of up to 2.5 gL^-1^h^-1^ (Argyros et al., 2011). This rate is faster than the commonly used combination of yeast and fungal enzymes (Holwerda et al., 2019). However, the ethanol production ability of this organism still requires improvement, as the maximum ethanol titer achieved so far is approximately 25–30 g/L (Holwerda et al., 2020; Hon et al., 2018; Tian et al., 2016), which is lower than the 40–50 g/L titers thought to be necessary for commercial application (Dien et al., 2003; Lynd et al., 2022; Matsakas et al., 2018; Zacchi and Axelsson, 1989).

A puzzling feature of *C. thermocellum* metabolism is that the rate of glycolysis slows dramatically at ethanol titers above 25 g/L (Tian et al., 2017b), and this is accompanied by an accumulation of hexose phosphates (Cui et al., 2020; Herrero et al., 1985; Tian et al., 2017b). It has also been observed that *C. thermocellum* has an atypical glycolysis, with several reactions using different cofactors from canonical glycolysis (Zhou et al., 2013). We have previously hypothesized that these observations may be related, and that the atypical glycolysis of *C. thermocellum* may operate closer to thermodynamic equilibrium than canonical glycolysis. This hypothesis is supported by both computational thermodynamic modeling (Dash et al., 2019) and ^13^C labeling experiments (Jacobson et al., 2020).

To increase the thermodynamic driving force of glycolysis, a series of genetic modifications were performed with the net result of changing the cofactor specificity of the phosphofructokinase (PFK) reaction from the native cofactor, pyrophosphate (PP_i_), to the heterologous cofactor, ATP (Hon et al., 2022). In theory, this should increase the thermodynamic driving force of glycolysis in two ways: one is because the ATP-linked PFK reaction is more thermodynamically favorable than the PP_i_-linked reaction based on a comparison of ΔrG’° values (Beber et al., 2022). A second is because the ATP/ADP ratio is, in many organisms, much larger than the PP_i_/P_i_ ratio (Noor et al., 2014) (although this has not yet been experimentally verified in *C. thermocellum*). This work was successful in increasing the thermodynamic driving force of glycolysis, but the effects were localized to reactions in the vicinity of the PFK reaction, and there was no effect on ethanol titer (Hon et al., 2022).

One factor which has been hypothesized to limit ethanol titer is PP_i_ production. Despite substantial effort, we have not yet identified the source that supplies the PP_i_ needed for glycolysis in *C. thermocellum* (Kuil et al., 2022; Schroeder et al., 2023; Zhou et al., 2013), and even in our strain engineered to use ATP as a cofactor for the PFK reaction, PP_i_ is still needed for the pyruvate phosphate dikinase (PPDK) reaction in glycolysis (Olson et al., 2017). We therefore constructed a strain where the PFK reaction uses ATP as a cofactor, and the PPDK reaction had been eliminated. The construction of this strain is described in more detail in a separate manuscript (Hon/Sharma, et al. 2024 - in preparation), however in the process of constructing this strain, we observed a mutation in the native *adhE* gene. The goal of this work is to understand the effects of that mutation in more detail and attempt to complement the deletion by expression of heterologous *adh* genes.

## 2 Methods

### 2.1 Alcohol dehydrogenase gene selection

The *adhB* gene from Z*ymomonas mobilis* is known to enable high titer ethanol production in that organism (Conway et al., 1987). However *Z. mobilis* is a mesophilic organism with a maximum growth temperature of 36 °C (Li et al., 2021). To identify enzymes similar to the *adhB* gene from thermophilic organisms, we searched the JGI IMG database (https://img.jgi.doe.gov/) (Markowitz et al., 2012). First, we identified genomes from thermophilic organisms. Using the Advanced Search Builder, we identified organisms whose “Temperature Range” field had the value of “Thermophile” or “Hyperthermophile.” We then searched these genomes for genes matching any of the following database identifiers: KEGG KO ID (Kanehisa et al., 2016) of K00001, K00002, or K13953, COG ID (Galperin et al., 2015) of COG1454 or COG1064. This resulted in a set of 100 genes. A subset of 18 of them were selected for synthesis and subsequent characterization (Table 1).

**Table 1:** List of genes expressed in *C. thermocellum* in this work showing the genome origin of each selected gene and its location in the genome (Locus Tag).

Organism	Growth Temperature (°C)	Growth Temp. Reference	Chromosome Accession Number	ADH Locus Tag
*Thermoanaerobacterium saccharolyticum*	45–70	Currie et al. (2015)	CP003184	Tsac0285
				Tsac1049
				Tsac2222
*Thermoanaerobacterium thermosaccharolyticum*	55–60	Litti et al. (2022)	CP002171	Tthe0864
				Tthe0472
*Thermoplasma acidophilum*	55–60	Segerer et al. (1988)	AL139299	Ta0858
				Ta1316
*Parageobacillus thermoglucosidasius*	40–60	Wada and Suzuki (2020)	CP002835	Geoth1941
				Geoth3826
				Geoth1917
*Thermoanaerobacter mathranii*	50–75	Larsen et al. (1997)	CP002032	Tmath0350
				Tmath1402
*Geobacillus thermodenitrificans*	50–70	Wada and Suzuki (2020)	CP000557	Gtng1754
*Thermotoga maritima*	55–90	Huber et al. (1986)	AE000512	Tm0920
*Thermococcus kodakarensis*	60–100	Atomi and Reeve (2019)	AP006878	Tk1008
*Zymomonas mobilis*	25–36	Li et al. (2021)	AE008692	Zmo1236
				Zmo1596 (*adhB*)
				Zmo1771

### 2.2 Strains and plasmids

Genes from Table 1 were synthesized and cloned into the pDGO143 plasmid backbone (Accession Number: KX259110.1)(Hon et al., 2016) by the U.S. Department of Energy Joint Genome Institute (JGI). Plasmids were prepared from *E. coli* cells where *dcm* methylation is not present (NEB C2566) to increase the efficiency of subsequent transformation into *C. thermocellum* (Guss et al., 2012).

**Fig. 1:**
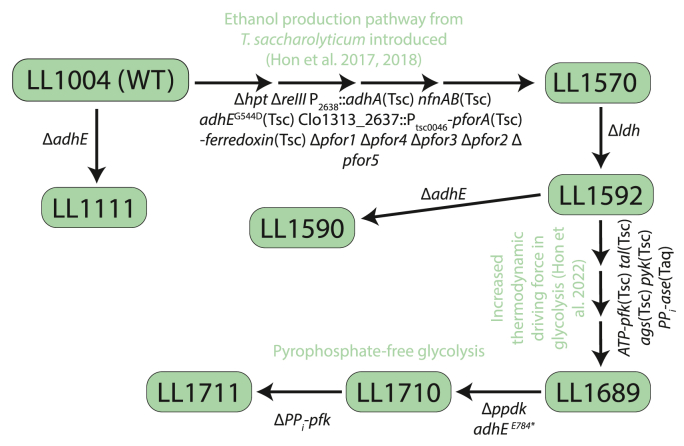
Diagram of genetic modifications used to produce the strain lineages described in this work.

Genome sequence data of strains described in this work are available from the NCBI Sequence Read Archive (SRA) database (https://www.ncbi.nlm.nih.gov/sra/). This sequence data serves as a complete description of the genotype of the relevant strains whose shorthand genotypes are listed in Fig. 1. A strain of *C. thermocellum* where glycolysis had been engineered for increased thermodynamic driving force (strain LL1711, SRA accession number SRX9409011) was used for fermentation assays to assess the ability of *adh* genes to increase ethanol production. This strain was derived from a lineage that included strain LL1570 (SRA accession number SRX4014213) (Hon et al., 2018), LL1592 (SRA accession number SRX5290154), LL1689 (SRA accession number SRX7724531), and LL1710 (SRA accession number SRX9409012). The relationship of these strains is shown in Fig. 1.

To measure enzyme activity, *adh* genes were expressed in an *adhE* deletion strain (LL1111, NCBI SRA accession number SRX744221) with lower background levels of ADH activity (Lo et al., 2015).

CTFUD rich medium (Olson and Lynd, 2012) was used for genetic manipulations and MTC-5 chemically defined medium (Hon et al., 2017) was used for fermentations.

### 2.3 Plasmids extraction

*E. coli* strains with plasmids previously synthesized by JGI (Table 1) were grown in 50 mL LB media with carbenicillin (10 μg/mL) and plasmids were extracted by the Monarch® Plasmid Miniprep Kit (New England Biolabds-NEB). Extracted DNA was stored in −20 °C to be further used for *C. thermocellum* transformation and sequencing.

### 2.4 Bacterial transformation

*C. thermocellum* transformation was performed as described by Olson and Lynd (2012). Cells were grown at 55 °C in 50 mL CTFUD until OD_600_ = 0.6 and harvested by centrifugation. They were washed with autoclaved ultrapure water three times and centrifuged again. The cell pellet was resuspended in ∼250 μL of water to form a concentrated cell suspension. 25 μL of this cell suspension was used for transformation with 0.5–1.0 μg of DNA. After transformation, cells were resuspended in 5 mL of CTFUD rich medium and grown overnight at 50 °C in a heat block. After growth, they were plated in CTFUD Agar medium with 6 μg/mL thiamphenicol and incubated at 55 °C. All of the steps of transformation, except centrifugation, were performed under anaerobic conditions in a Coy anaerobic chamber (Coy Laboratory Products, Grass Lakes, MI).

### 2.5 Fermentations and product quantification

For routine cultivation, *C. thermocellum* strains were grown anaerobically at 55 °C in a Coy Anaerobic chamber with gas phase of 85% N_2_, 10% CO_2_ and 5% H_2_.

For *adh* gene expression studies, colonies were collected from transformation plates and inoculated in 2 mL tubes containing 1.5 mL of MTC-5 medium with 30 g/L cellobiose and 6 μg/mL thiamphenicol (Hogsett, 1995; Hon et al., 2017; Sharma et al., 2023). They were incubated at 55 °C in the same medium for 4 days. The pDGO143 plasmid was used as an empty-vector control.

For strains LL1592, LL1689, LL1710 and LL1711, batch fermentations were performed in MTC-5 medium with 60 g/L cellobiose in serum bottles with headspace purged with 100% nitrogen as previously described (Hogsett, 1995; Hon et al., 2017; Sharma et al., 2023). These cultures were incubated at 55 °C for 7 days.

Fermentation products were quantified by Waters™ HPLC (Milford, MA) with refractive index and UV detector, having an Aminex HPX-87H column (Bio-Rad, Hercules, CA) as previously described (Holwerda et al., 2014).

### 2.6 Alcohol dehydrogenase assays

ADH activity was measured as described by Olson et al. (2023). The reaction mixture contained 100 mM Tris-HCl buffer, 0.24 mM NADH or NADPH, 1 mM DTT and various amounts of cell extract. Each cell extract was measured at 6 different 3-fold serial dilutions. NAD(P)H consumption was observed by decrease in the 340 nm absorbance after addition of 17.6 mM of acetaldehyde to the reaction mixture. The assay was performed in a Coy anaerobic chamber at 40 °C and pH 7.0. Assays were performed in a 384-well plate, with a 0.67 cm pathlength, a 60 μL total volume and measured in an Agilent Biotek Epoch 2 spectrophotometer (Olson et al., 2023).

### 2.7 Neighbor-joining and functional protein analysis

Both Neighbor-Joining (NJ) and functional protein analysis were conducted with AdhE protein sequences in Geneious Prime® 2023.2.1 (https://www.geneious.com) software. The NJ distance analysis (Saitou and Nei, 1987) and sequence divergences were calculated with the Jukes-Cantor model. Node supports were measured by 1000 replicates of bootstrap. Protein domains were predicted using Interproscan 2.1 plugin to search for protein families (Pfam's) and superfamily (Finn et al., 2015).

## 3 Results and discussion

### 3.1 Parental strains background and fermentation profile

**Fig. 2:**
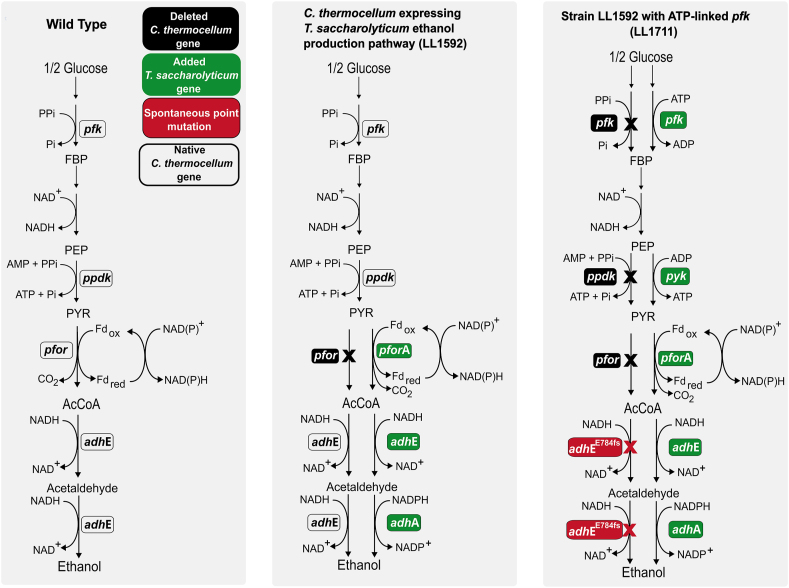
Graphical explanation of *C. thermocellum* strains used in this work. White boxes represent native *C. thermocellum* genes, black boxes represent knockouted genes, green boxes represent heterologous expressed *T, saccharolyticum* genes and red boxes indicate spontaneous mutation occurred in strain LL1711. X sign represents inactivated reactions. Abbreviations: FBP - Fructose - 1,6 - bisphosphate; PEP - Phosphoenolpyruvate; PYR - Pyruvate; AcCoA - Acetyl-CoA; *pfk* - Phosphofructokinase; *ppdk* - Pyruvate phosphate dikinase; *pfor -* Pyruvate ferredoxin/flavodoxin oxidoreductase; *adh* - Alcohol dehydrogenase. (For interpretation of the references to colour in this figure legend, the reader is referred to the Web version of this article.)

Strain LL1570 was created by introducing the *T. saccharolyticum* ethanol production pathway into *C. thermocellum*, with the purpose of increasing ethanol yield (Hon et al., 2017, 2018). Subsequent deletion of the lactate dehydrogenase gene (*ldh*) resulted in strain LL1592 (Hon et al., 2022). Strain LL1711 builds upon strain LL1592 and contains substantial genetic modification with the goal of improving the thermodynamic driving force of glycolysis, incorporating lessons learned from the first attempt (Hon et al., 2022). In the resulting strain several heterologous genes from *T. saccharolyticum* are expressed, including *adhE, adhA, pforA, pyk and pfk*. The strain also contains deletion of several native *pfor genes* and the PPi-linked *pfk* gene (see Fig. 2)*.* The details of development of this strain will be published in a separate manuscript (Hon/Sharma, et al. 2024 - in preparation).

**Fig. 3:**
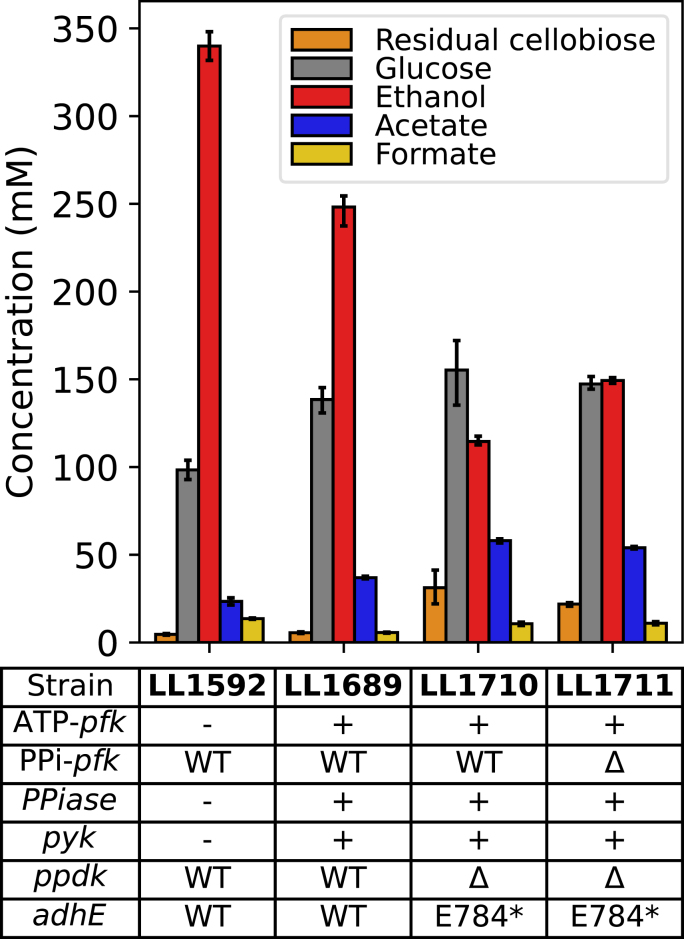
Fermentation product profiles for *C. thermocellum* strains (LL1592, LL1689, LL1710 and LL1711) grown in MTC-5 media with 60 g/L cellobiose as substrate. The ‘+' sign indicates introduction of heterologous gene, ‘-’ sign indicates absence of heterologous gene, ‘WT’ indicates wildtype gene, ‘Δ’ indicates deletion of wild type gene, and 'E784*’ indicates nonsense mutation present in *C. thermocellum* AdhE protein at position 784. Error bars represent 1 standard deviation (n ≥ 2).

Strain LL1711 acquired an inactivating E784* mutation in the native *adhE* gene, which resulted in decreased ethanol yield and titer versus strain LL1592 (Fig. 3). However this strain still produces ethanol, presumably due to the presence of ALDH activity from the *T. saccharolyticum adhE* gene (note that the strain has two *adhE* genes--the native one and a heterologous one from *T. saccharolyticum*). We were therefore interested to understand whether the inactivation of the native *adhE* gene could be complemented to increase ethanol production. Initially, we attempted to do this via plasmid-based expression of the WT *C. thermocellum adhE* gene, however transformation was not successful (data not shown). Subsequently, we attempted complementation with monofunctional *adh* genes.

### 3.2 Fermentations profile of *C. thermocellum* strains expressing monofunctional alcohol dehydrogenases

In order to compensate for the decrease in ADH activity in strain LL1711, 18 monofunctional *adh* genes were heterologous expressed in this strain (Table 1). This set of genes included the *adhB* gene from *Z. mobilis* (locus tag Zmo1596), which is known to play a key role in the high ethanol titers reported by that organism (Tamarit et al., 1997; Yomano et al., 2008). It also included several others chosen for similarity to the *adhB* gene. Since *Z. mobilis* is a mesophilic organism, chromosomes of thermophilic organisms from the JGI IMG database (https://img.jgi.doe.gov/) were searched to identify candidates likely to have high thermostability.

**Fig. 4:**
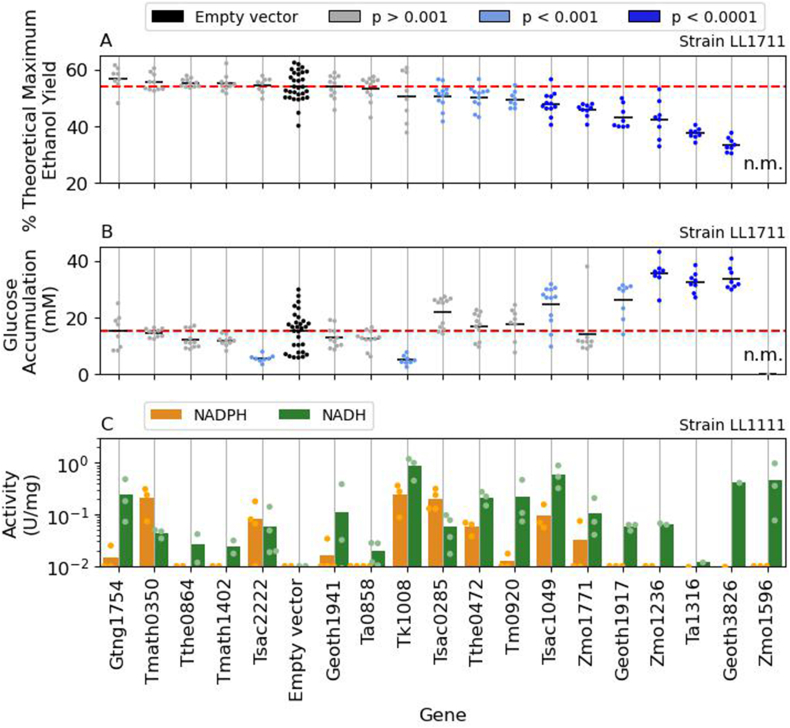
Effect of *adh* gene expression on ethanol production. Adh genes were cloned in the pDGO143 plasmid backbone (empty vector) and expressed in a *C. thermocellum* strain engineered for increased ethanol production (strain LL1711, several modifications) and non ethanol producer strain (strain LL1111) for enzymatic assays. (A) % Theoretical ethanol yield obtained by engineered strains and (B) Glucose accumulation in media after 96 h fermentation at 55 °C, anaerobically, in MTC-5 media 30 g/L cellobiose (88,2 mM). (C) Specific activity of ADH enzymes for either NADPH (orange) or NADH (green) cofactors. Each dot represents a different colony expressing the respective gene. Red dotted lines indicate mean values for control strain. Statistical significance was calculated using a one-way ANOVA test. p < 0.05 was considered significant. Limit of detection of activity by the equipment was of 10^-3^ for NADH and 10^-2^ for NADPH. The *adhB* gene from *Z. mobilis* corresponds to the Zmo1596 locus tag. The complete fermentation data used to generate panels A and B is included in Supplementary Table S1. The complete enzyme assay data used to generate panel C is included in Supplementary Table S2. The absence of data is indicated by the initials n.m. (not measured). (For interpretation of the references to colour in this figure legend, the reader is referred to the Web version of this article.)

After a four-day fermentation, the majority of engineered strains were able to consume all of the initial cellobiose substrate (Supplementary Table S1), and ethanol yield remained within the range of control strain (Empty Vector) (Fig. 4A and B). Although none of the strains showed a significant *increase* in ethanol production, several showed a significant *decrease* in ethanol production. Interestingly, the *adhB* gene from *Z. mobilis* was not able to be transformed into strain LL1711, and thus ethanol production for that enzyme was not measured (n.m.). Strains with decreased ethanol yield showed an increase in glucose accumulation. Since cellobiose can be degraded to glucose extracellularly, strains with high levels of glucose accumulation may represent cessation of metabolism. We did not observe any other significant changes in other measured metabolites (formate, acetate, lactate, malate, pyruvate, or succinate). All of the fermentation data for all strains (including carbon balances) is presented in Supplementary Table S1.

To better understand why some strains exhibited lower ethanol titers, we measured enzyme activity in cell lysates. Since several heterologous *adh* genes are expressed in strain LL1711, we performed the enzyme activity measurements in an *adhE* deletion strain (strain LL1111) with lower ADH background activity. We were able to successfully transform all of the *adh* expression constructs into this strain (including the *adhB* gene from *Z. mobilis*). We then measured ADH activity with both NADH and NADPH cofactors (Fig. 4, panel C). Although it is difficult to draw strong conclusions, we did notice that strong NADH-linked ADH activity was overrepresented in strains with decreased ethanol yield. We also tested enzyme reversibility, but did not see any notable differences between forward and reverse activity among any of the Adh enzymes tested (data not shown). It has been shown in several organisms that eliminating NADH-linked ADH activity increases ethanol tolerance (Brown et al., 2011; Burdette et al., 2002; Lovitt et al., 1988; Olson et al., 2023; Tian et al., 2019), and this is presumed to be due to the sensitivity of the glyceraldehyde-3-phosphate dehydrogenase reaction to high ratios of NADH to NAD^+^ (Lovitt et al., 1988; Tian et al., 2017b).

There are many examples of organisms with multiple Adh enzymes (Carere et al., 2012; de Smidt et al., 2008; Ismaiel et al., 1993; Radianingtyas and Wright, 2003; Reid and Fewson, 1994). Although in yeasts and ethanol-producing bacteria, it is known that some Adh enzymes have distinct roles in ethanol production vs. uptake (Denis et al., 1983; de Smidt et al., 2008; Neale et al., 1986), the physiological role of multiple Adh enzymes in other organisms is often difficult to determine. In many organisms, deletion of *adh* genes results in decreased ethanol production (Brown et al., 2018; Ida et al., 2012; Lo et al., 2015; Rodriguez and Atsumi, 2012, 2014; Yao and Mikkelsen, 2010). However there are some exceptions. For example, the thermophilic anaerobe *Thermoanaerobacter ethanolicus* contains at least three Adh enzymes (AdhE, AdhA, and AdhB), and deletion of either AdhA or AdhB (but not both) allowed increased ethanol yield (Zhou et al., 2017). There are only isolated examples of comparison of the effect of overexpression of Adh isoenzymes. In yeast, overexpression of the ADH2 gene (thought to be primarily responsible for ethanol oxidation) had no effect on ethanol production (Maestre et al., 2008).

### 3.3 Neighbor-joining and functional protein analysis

**Fig. 5:**
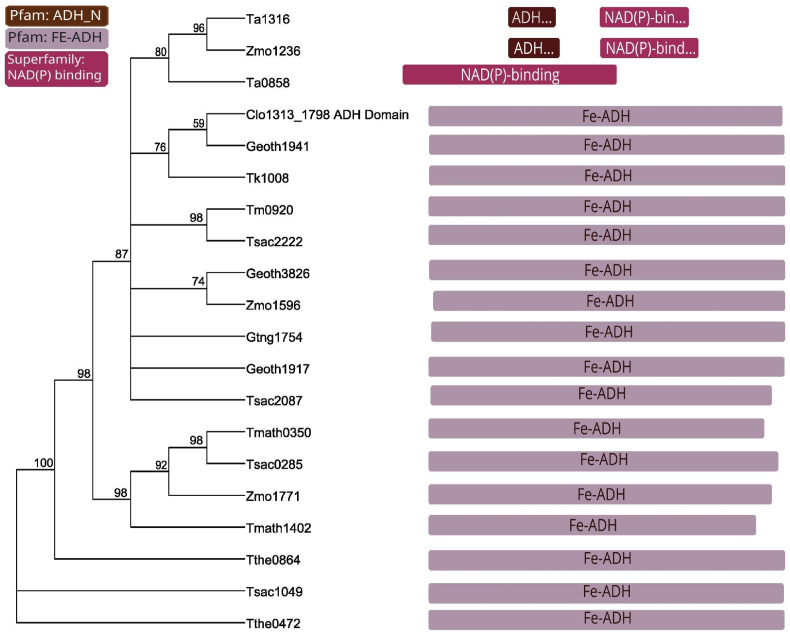
Neighbor-joining consensus tree inferred using ADH protein sequences. Node numbers refer to NJ bootstrap proportions (in percentage) among 1000 replicates. Node supports values below 70% were not recorded in the tree. Boxes on the right represent the domains of each protein, being the brown and purple boxes Pfam domains and pink the superfamily. (For interpretation of the references to colour in this figure legend, the reader is referred to the Web version of this article.)

Next, we investigated whether the different effects on ethanol yield were correlated with sequence similarity. We performed both (i) neighbor-joining analysis and (ii) analyzed patterns in the amino acid sequence using the Pfam database (Finn et al., 2015) (Fig. 5). We saw only a few relatively weak patterns here as well. The Adh protein with the lowest ethanol yield was Geoth3826, which was most similar to Zmo1596 (*adhB*), which could not be transformed into strain LL1711. The Ta1316 and Zmo1236 proteins both share a relatively distinct domain structure, and also reduced ethanol yield.

### 3.4 Conclusions

Here we screened several monofunctional Adh enzymes and identified several that are functionally expressed in *C. thermocellum*. Interestingly, enzymes from the mesophile *Z. mobilis* were highly active in *C. thermocellum* even at the thermophilic temperatures used in this work (55 °C).

The genes characterized in this work might be useful in the future for ethanol production pathways that use the pyruvate decarboxylase (Pdc) enzyme instead of pyruvate ferredoxin oxidoreductase (Pfor) enzyme. Since the Pdc enzyme produces acetaldehyde, it may benefit from co-expression with monofunctional Adh enzymes (i.e. as opposed to AdhE, which is a bifunctional enzyme). Previously we have shown that the Pdc pathway benefits from coexpression of a monofunctional AdhA enzyme (Tian et al., 2017a), and the additional Adh enzymes characterized in this work provide further alternatives.

We observed an unexpected result: that increased Adh expression never led to increased ethanol production and in several cases actually *decreased* it. This suggests that ethanol production in strain LL1711 is not limited by the ADH activity, and provides some support to our hypothesis that NADH-linked ADH activity may be incompatible with high titer ethanol production. However the mechanisms behind this remain to be explored.

## Funding

This work was supported by São Paulo Research Foundation (FAPESP) process number 18/25682-0. DPC was financially supported by the São Paulo Research Foundation (FAPESP) with a PhD scholarship (process number 2021/10434-4) and a BEPE scholarship (process number 2022/03642-2).

This work was partly supported by the Center for Bioenergy Innovation (CBI), 10.13039/100000015U.S. Department of Energy, 10.13039/100006132Office of Science, 10.13039/100006206Biological and Environmental Research Program under Award Number ERKP886.

Synthesis of DNA constructs was conducted by the U.S. Department of Energy Joint Genome Institute (https://ror.org/04xm1d337), a DOE Office of Science User Facility, is supported by the Office of Science of the U.S. Department of Energy operated under Contract No. DE-AC02-05CH11231.

## CRediT authorship contribution statement

**Daniela Prates Chiarelli:** Writing – review & editing, Writing – original draft, Validation, Methodology, Investigation, Data curation. **Bishal Dev Sharma:** Writing – review & editing, Visualization, Investigation. **Shuen Hon:** Writing – review & editing, Visualization, Resources, Investigation. **Luana Walravens Bergamo:** Writing – review & editing, Supervision. **Lee R. Lynd:** Writing – review & editing, Supervision, Funding acquisition. **Daniel G. Olson:** Writing – review & editing, Writing – original draft, Visualization, Validation, Supervision, Methodology, Investigation, Funding acquisition, Data curation, Conceptualization.

## Declaration of competing interest

The authors declare the following financial interests/personal relationships which may be considered as potential competing interests: One co-author, Lee R. Lynd, is the CEO of the Terragia corporation, which has a financial interest in commercialization of processes involving *Clostridium thermocellum*.

## Data Availability

Data will be made available on request.
